# Mitigating aviation’s environmental impact through alternative fuels and cleaner engines

**DOI:** 10.1126/sciadv.aeg7503

**Published:** 2026-09-18

**Authors:** Rebecca Dischl, Daniel Sauer, Christiane Voigt, Raphael Märkl, Gregor Neumann, Tiziana Bräuer, Theresa Harlaß, Stefan Kaufmann, Monika Scheibe, Anke Roiger, Katharina Seeliger, Charles Renard, Amandine Roche, Georg Eckel

**Affiliations:** ^1^ Deutsches Zentrum f. Luft- und Raumfahrt, Insitut f. Physik der Atmosphäre, Oberpfaffenhofen, Germany.; ^2^ Johannes Gutenberg University, Institute of Atmospheric Physics, Mainz, Germany.; ^3^ Airbus Operations SAS, Toulouse, France.; ^4^ Safran Aircraft Engines, Villaroche, France.; ^5^ Deutsches Zentrum f. Luft- und Raumfahrt, Institute of Combustion Technology, Stuttgart, Germany.

## Abstract

Lean-burn engines are designed to reduce emissions, and sustainable alternative fuels provide additional potential for lowering particle emissions. However, the magnitude of the benefits from these mitigation levers remains poorly constrained, limiting robust predictions of aviation’s climate impact. We present in-flight measurements of particle emissions from modern lean-burn LEAP-1A engines operated on Airbus A319neo and A321neo aircraft, burning conventional and sustainable aviation fuels. Emissions of nonvolatile particles larger than 14 nanometers from lean-burn engines were reduced by three orders of magnitude compared to rich-burn operation. Under rich-burn conditions, soot particle number emissions show an exponential dependence on fuel hydrogen content, with engine-specific variations. Volatile particle emissions scale with fuel sulfur content, and substantial differences were observed even between two engines of the same type. Optimizing fuel composition and combustor technology may reduce particle emissions, improve airport air quality, and mitigate contrail radiative forcing.

## INTRODUCTION

Aircraft emissions play a critical role in the environmental impact of aviation, influencing Earth’s radiative energy budget and degrading air quality (*1*). Particulate matter (PM) is of particular concern due to its potential effects on human health and complex interactions with the atmosphere (*2*–*4*). Under certain atmospheric conditions, particles emitted by aircraft engines lead to the formation of contrails, which are substantial contributors to radiative forcing of aviation. Contrails can evolve into larger-scale cirrus clouds (*5*–*7*), trapping outgoing longwave radiation. The effective radiative forcing (ERF) from contrails is comparable to or can even exceed the warming ERF of carbon dioxide emissions from aviation (*1*, *8*, *9*). Uncertainties in the radiative effects of contrails are multifold and large, on the order of ±70% (*1*), and recent studies address parts of those ambiguities (*10*–*12*). In addition, a recent risk analysis, which is based on the global warming per activity metric (i.e., the integrated ERF over 1 year of flight operations) and incorporates uncertainty distributions, suggests that mitigation of aviation’s non-CO_2_ effects may be favored in certain trade-off situations for CO_2_–to–non-CO_2_ ratios smaller than 1:5 (*13*, *14*).

Contrails form when the hot aircraft exhaust gases are cooled by mixing with the cold atmospheric air at cruise altitude. If the ambient temperature is below the so-called Schmidt-Appleman threshold temperature (*T*_SA_), then the exhaust plume reaches liquid supersaturation. Then, water vapor condenses onto emitted, newly formed, or preexisting atmospheric volatile and nonvolatile aerosols, forming droplets that quickly freeze into ice particles (*15*). For current rich-burn engines, the soot particles produced during combustion serve as the primary source of condensation nuclei for the water droplets (*16*, *17*). Recent studies (*18*–*21*) have shown that the soot-to-ice correlation can be modulated by the fuel sulfur content, which can increase or decrease contrail ice crystal numbers.

The development and use of sustainable aviation fuels (SAF), for example, HEFA-SPK (hydroprocessed esters and fatty acid synthetic paraffinic kerosene), as well as biofuel blends, can reduce the life cycle CO_2_ footprint and particulate emissions: Previous studies have demonstrated that the use of SAF and SAF blends in classical rich-quench-lean (RQL), or rich-burn, engines with soot emission indices (EIs) in the order of 10^14^ to 10^15^ per kilogram fuel burned can reduce soot emissions (*22*, *23*), which decreases ice crystal numbers (*18*, *21*, *24*, *25*) and the climate forcing of contrails (*8*, *26*). The composition, size, and number concentration of the volatile and nonvolatile exhaust aerosols can alter contrail ice crystal numbers and the optical properties, lifetime, and therefore the ERF of contrail cirrus clouds (*8*, *9*, *27*, *28*).

Technological advancements in engine design, such as modern lean-burn and advanced RQL combustors, aim to further reduce aircraft emissions. In lean-burn combustors, the fraction of the combustor volume operating at lean fuel-to-air ratios is maximized through differences in air-fuel distribution and combustor design compared to rich-burn combustors, potentially leading to lower NO*_x_* and soot particle emissions, as confirmed during ground engine tests (*29*). Also, lower soot particle emissions have been found close to airports (*30*). However, the influence of lean-burn engines on particle emissions and their characteristics at cruise altitude have hardly been researched yet. Contrail ice particle numbers of the same order of magnitude were observed for an engine operated in lean and forced rich combustion conditions (*31*), raising the question of which aerosols serve as condensation nuclei for current lean-burn engine configurations.

Aircraft emissions are typically characterized from ground-based tests, but in contrast to NO*_x_* emissions, large uncertainties persist when extrapolating such measurements to cruise conditions for nonvolatile particulate matter (nvPM), volatile aerosol precursors, and the resulting volatile particles, whose formation depends on conversion efficiencies and ambient conditions (*23*). This gap constrains in-flight engine nvPM modeling based on ground measurement data, such as those in the ICAO Aircraft Engine Emissions Databank (*29*). This limitation is even more pronounced for vPM, which are not part of the engine certification testing. Further, the influence of the fuel composition in terms of its hydrogen and sulfur content on particle emissions in rich-burn and lean-burn combustor conditions has hardly been explored in flight experiments. Both dependencies are required to predict aviation’s climate impact and to assess mitigation measures. The in-flight measurements presented here help close this gap by examining the characteristics of particle emissions from modern aircraft engines burning alternative fuels and conventional Jet A-1 under typical operating conditions. By comparing particle emissions from different aviation fuels and aircraft engines, our findings enhance the understanding of aircraft emissions in the low-soot regime during cruise and serve as valuable input for advancing model development. Moreover, the results may support the optimization of future engine technologies for lower particle emissions, facilitate compliance with evolving regulations, and thereby help to reduce the impact of aviation on climate and public health.

## RESULTS

### Experiment and instrumentation

Measurement data were gathered as part of the NEOFUELS/VOLCAN (Compatibilité et Vol Carburants Alternatifs Nouveaux) campaign series (*31*), conducted in November 2021 (step 1) and March 2023 (step 2). The flight experiments aimed to characterize cruise emissions of CFM LEAP-1A engines (see the “Source aircraft engines” section) and to assess the influence of the combustion mode (i.e., lean-burn versus rich-burn) and different jet fuels on aerosol and trace gas emissions of the test engines as well as contrail ice particles. An Airbus A319neo (reg. D-AVWA) served as the source aircraft for step 1, and an Airbus A321neo (reg. D-AVZO) served as the source aircraft for step 2. Additional background information and insights into the campaign operations are provided by Voigt *et al.* (*31*, *32*).

For the specific purposes of the flight test campaigns, a FADEC (Full Authority Digital Engine Control) engine control trim was defined to allow for continuous operation in a forced rich-burn mode, whereas in typical fleet operation, lean-burn mode would be automatically commanded. This temporary operating mode enriches the fuel-air mixture beyond the typical lean configuration by adjusting fuel flow and air mixing. However, it differs from the classical rich-burn phase of an RQL combustor, mainly due to differences in air distribution.

The engine power setting was defined via the combustor inlet temperature (T30), which was fixed at a constant temperature typical for cruise flight conditions at the time of the measurement. For near-field measurements, the probed engine of the source aircraft was operated at typical cruise settings, while the thrust of the other engine was adapted to operate at the same altitude and speed as the DLR Falcon measurement aircraft. Because of speed limitations of the measurement aircraft, measurements were not conducted at the typical cruise speed of the source aircraft. In this context, the effect of aircraft speed is primarily reflected in a reduced combustor inlet pressure (P3), while T30 remains fixed at cruise conditions. The corresponding impact of the combustor inlet pressure variation can be estimated using the pressure coefficient (*P*3_flight_/*P*3_ground_)^1.35^ of the MEEM v1 methodology (*33*), yielding an expected reduction in soot emissions of less than 5% when considering the speed-related effect alone.

Comparisons of particle emissions from different fuels and combustion modes were always conducted behind the right-hand engine. In addition, an engine-to-engine intercomparison was performed. Absolute values for T30 and fuel flow fall under intellectual property of the engine manufacturer and, therefore, are not disclosed.

During the measurements, a conventional Jet A-1 fuel obtained from the local supplier at Toulouse Blagnac airport (TotalEnergies) was used as reference fuel. Compared to global average Jet A-1 (*34*, *35*), the reference Jet A-1 supplied for the campaign had a higher hydrogen content, a lower carbon and sulfur content, as well as reduced aromatics and naphthalenes (see Table 1), indicative of cleaner fuel properties. During NEOFUELS/VOLCAN step 1, the conventional fuel was compared with 100% HEFA-SPK. For NEOFUELS/VOLCAN step 2, the conventional fuel was compared with a fuel blend composed of 19% Jet A-1 and 81% HEFA-SPK, as well as with two HEFA-SPKs with added aromatics. The added aromatics consisted of alkylbenzenes of fossil origin, with a majority of C9 compounds (i.e., alkylbenzenes containing nine carbon atoms; ∼60%), as well as C10 (10 carbon atoms; ∼25%) and C8 compounds (eight carbon atoms; ∼15%). This composition was chosen with the aim of being similar to synthetic aromatics (*36*). One fuel had a lower aromatic content (SPK + LA), and the other fuel had a higher aromatic content (SPK + HA) than the reference Jet A-1 to allow investigation of the influence of the fuel aromatic content on particle emissions and contrails. After production, both fuels were almost sulfur free, but traces of sulfur were found in the samples taken from the aircraft tanks. This can be attributed to contamination in the supply chain and aircraft tanks, resulting in a sulfur content of 9 parts per million by mass (ppm) for the SPK + LA and 4 ppm for the SPK + HA. The engines of the A319neo and the A321neo can be fed from separate tanks, allowing to test two different fuels during a single flight under comparable atmospheric conditions.

**Table 1.** Selected properties of the tested fuels. The hydrogen content was analyzed according to ASTM standard D3701 with a repeatability of 0.09 and a reproducibility of 0.11, the sulfur content was analyzed according to ASTM standard D5453 with a detection limit of 1 ppm by mass, the aromatics content was analyzed according to ASTM standard D6379, and the naphthalene content was analyzed according to ASTM standard D1840. The carbon content refers to the difference between 100% and hydrogen content. Fuel samples were collected from the aircraft tank before the flights. In addition, differing FSC measured in fuel probes from the refueling truck are given in parentheses. Values reported for HEFA-SPK, SPK + LA, SPK + HA, and blend fuel refer to samples corresponding to the respective measurement flight, whereas values for Jet A-1 represent the average over all samples.

Fuel	Components	Hydrogen content (%m/m)	Carbon content (%m/m)	Sulfur content (ppm by mass)	Total aromatics (%v/v)	Naphthalenes (%v/v)
NEOFUELS/VOLCAN step 1						
Jet A-1	Conv. Jet A-1	14.2	85.8	138	13.1	0.4
HEFA-SPK	100% HEFA-SPK	15.3	84.7	6 (3)	<1	<0.1
NEOFUELS/VOLCAN step 2						
Jet A-1	Conv. Jet A-1	14.1	85.9	195	12.8	0.6
SPK + LA	91.6% HEFA-SPK + 8.4% aromatics	14.8	85.2	9 (2)	8.4	<0.1
SPK + HA	82.4% HEFA-SPK + 17.6% aromatics	14.3	85.7	4 (<1)	17.6	<0.1
Blend	19% Jet A-1 + 81% HEFA-SPK	15.1	84.9	41	2.5	0.2

In situ in-flight measurements were performed using the DLR-operated Dassault Falcon 20-E5 (Reg. D-CMET). The Falcon was equipped with various instruments for measuring aerosol particles and trace gases, with sampling conducted through inlets located along the upper fuselage centerline. The instruments measured the number of total particles larger than 5 nm (tPM), nonvolatile particles larger than 14 nm (nvPM), CO_2_, CO, and H_2_O, as well as the sum of NO*_x_* and all reactive nitrogen species (NO*_y_*). In addition, two cloud particle probes were mounted in underwing pods to detect ice particle number and size distributions in ambient atmospheric conditions. For details, see the “Aerosol and trace gas measurements” section. This study primarily focuses on aerosol measurements.

**Fig. 1.**
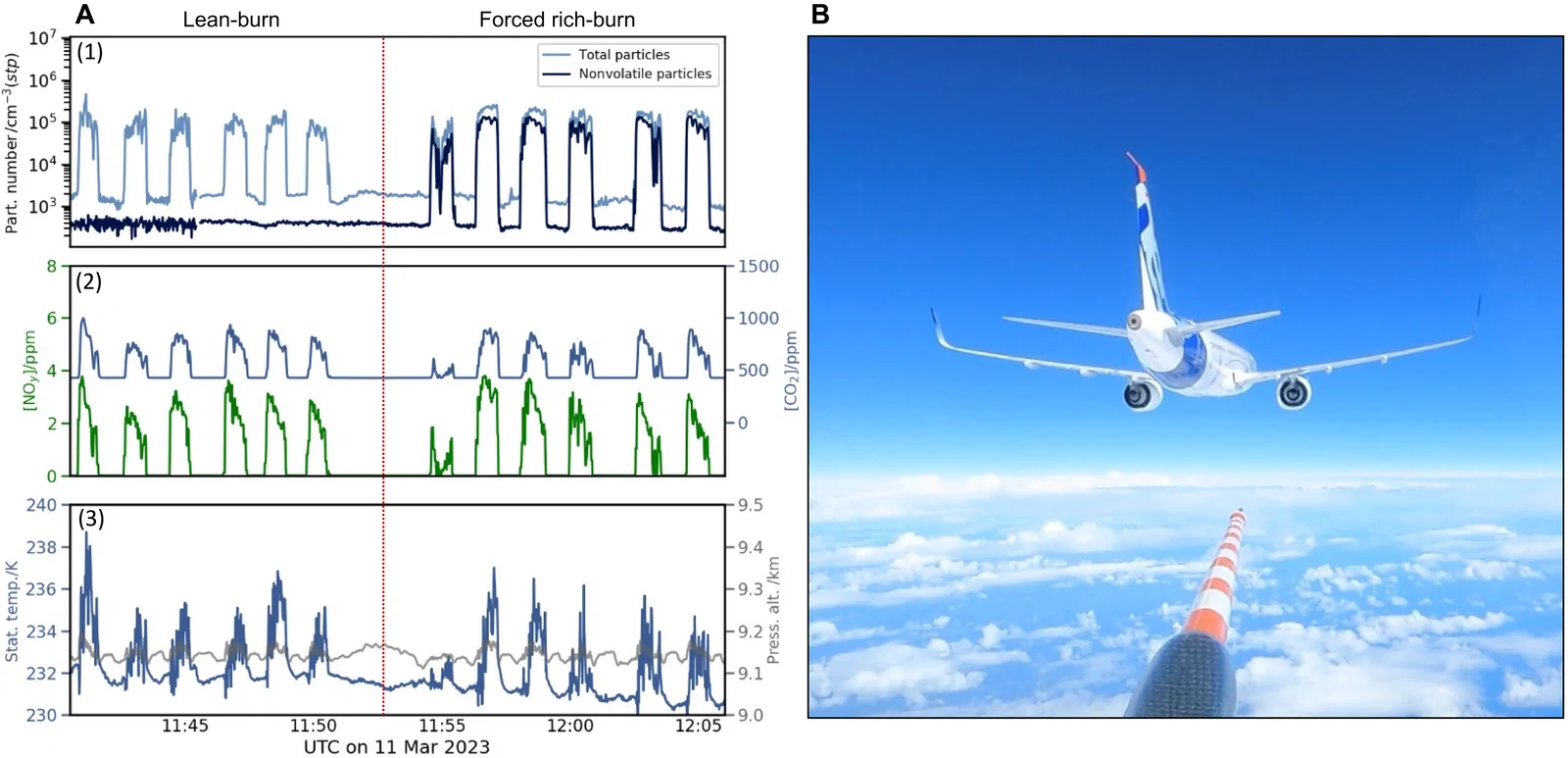
Near-field emission measurements during NEOFUELS/VOLCAN step 2. (**A**) Timeline of a near-field test sequence in lean-burn and forced rich-burn mode. Panel (1) shows number concentrations of total particles larger than 5 nm (light blue) and nonvolatile particles larger than 14 nm (dark blue) at standard temperature and pressure, panel (2) shows trace gas concentrations of NO*_y_* (green) and CO_2_ (blue), and panel (3) shows the altitude of the research aircraft as well as the ambient temperature. Peaks in the concentration signal represent the individual plume crossings. At 11:45 coordinated universal time (UTC), the dilution system for the particle measurements was switched off; at 11:53 (dotted red), the combustion mode was changed from lean-burn to forced rich-burn mode. Markedly lower nonvolatile soot particle emissions were measured during the lean-burn combustion operation. (**B**) photo from the Falcon cockpit chasing the A321neo during an emission measurement sequence.

The measurement flights followed racetrack patterns within restricted airspaces along the French Mediterranean and Atlantic coasts. Exhaust emissions were measured in the near field ∼30 to 250 m behind the source aircraft, corresponding to plume ages of 0.1 to 1.4 s, in areas where high ambient temperatures and low relative humidity prevented contrail ice particle formation, allowing for the measurement of unprocessed emissions. Figure 1 shows a time series from a near-field measurement flight on 11 March 2023, including particle and trace gas emissions, altitude, and ambient air temperature. As the Falcon ascended into the exhaust plume, distinct increases in particle and trace gas concentrations were observed. Typically, the Falcon approached the plume from the right-hand side and from slightly below to ensure that the fuselage-mounted inlets were well within the plume. Each near-field encounter lasted around 45 s, after which the Falcon descended to sample background concentrations. This process was repeated three to five times per test point to get sufficient data statistics.

### Impact of fuel and combustion mode on particle emissions

To evaluate the influence of different fuel properties and combustion modes on particle emissions for a specific engine condition, near-field measurements were performed at air temperatures above *T*_SA_ and relative humidity below 70%. This ensures that no contrail ice particles are formed that could influence exhaust aerosols. The sampled aircraft flew with a Mach number of 0.59 to 0.62 to allow the Falcon to match the flight speed. Measurement conditions are summarized in table S2. The combustor inlet temperature T30 of the probed engine was kept at typical cruise values, while the other engine was regulated to allow for the reduced cruise speed.

**Fig. 2.**
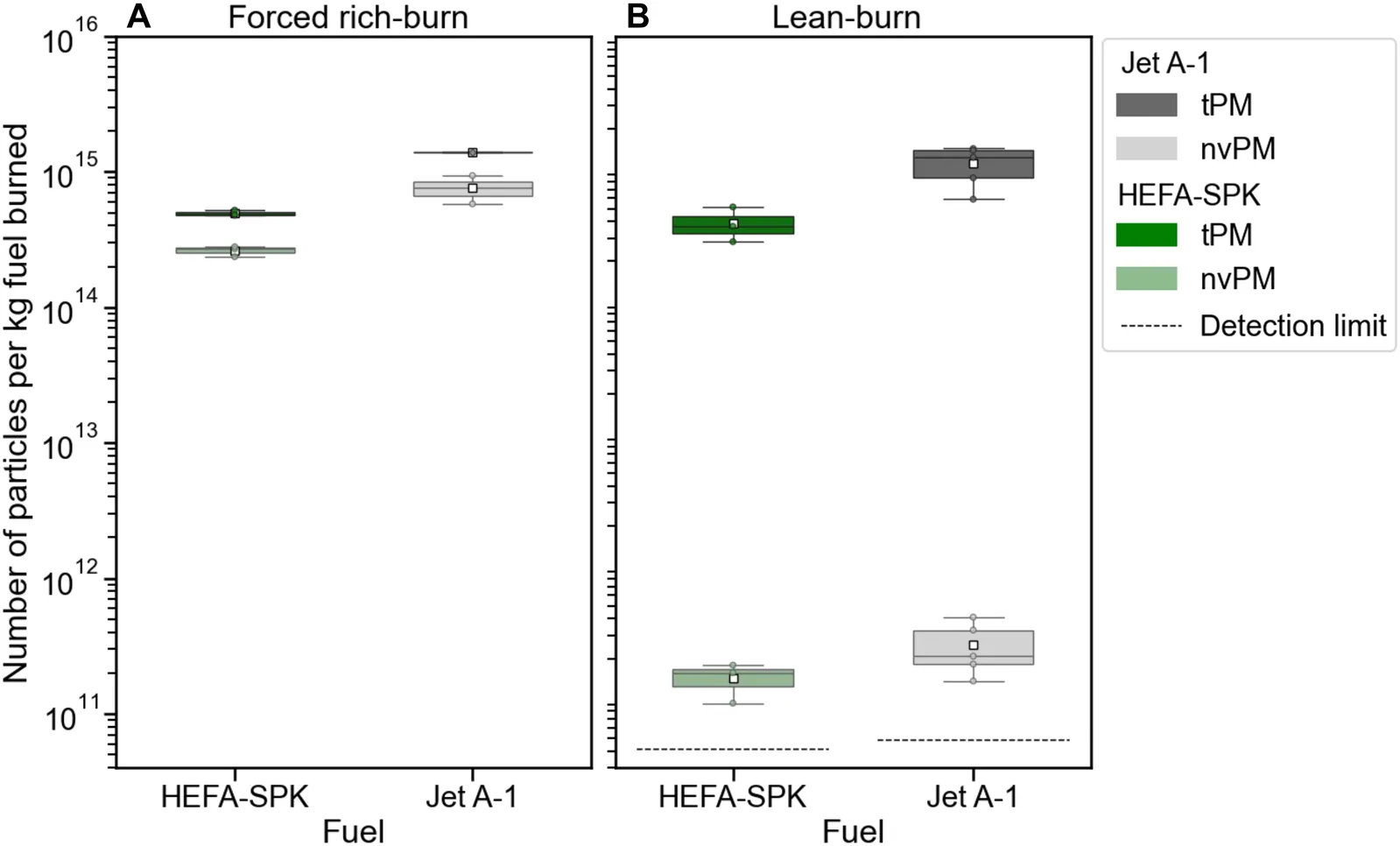
Near-field measurements of total particles >5 nm (top boxes) and nonvolatile particles >14 nm (bottom boxes) of the A319neo during NEOFUELS/VOLCAN step 1. Emissions of HEFA-SPK (green) and Jet A-1 fuel (gray) were measured at 9140-m flight altitude (FL300) in (**A**) forced rich-burn and (**B**) lean-burn combustion modes. Boxes extend from the first quartile to the third quartile of the data, with a horizontal line indicating the median and white squares indicating the mean. The whiskers extend to the furthest data point that falls within 1.5 times the interquartile range. Any data points that exceed the whiskers are considered outliers.

**Fig. 3.**
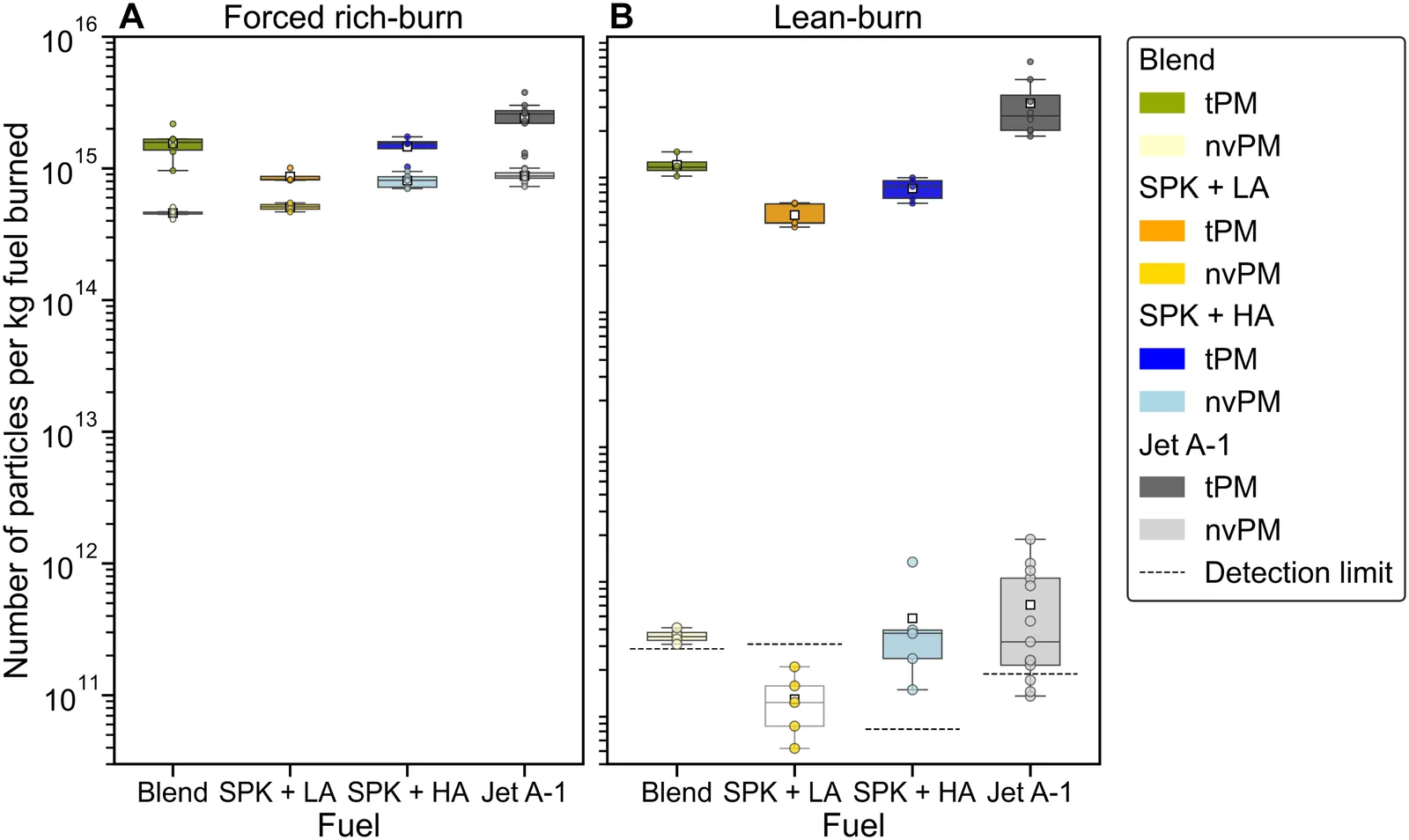
Near-field measurements of total particles >5 nm (top boxes) and nonvolatile particles >14 nm (bottom boxes) of the A321neo during NEOFUELS/VOLCAN step 2. Emissions of HEFA-SPK with low aromatic content (orange) and high aromatic content (blue), a blended fuel containing 19% Jet A-1 + 81% HEFA-SPK (green), as well as Jet A-1 fuel (gray) were measured in (**A**) forced rich-burn and (**B**) lean-burn combustion mode. EI_nv_ of SPK + LA from lean combustion were mostly below the detection limit, which increases the uncertainty. To indicate possible trends, statistics of these data are shown as blank box.

Figure 2 shows particle number EIs of the A319neo test aircraft operating in forced rich-burn mode (Fig. 2A) and lean-burn mode (Fig. 2B) burning 100% HEFA-SPK and the reference Jet A-1 from NEOFUELS/VOLCAN step 1. Similar reductions were presented in (*31*). Lean combustion mode substantially lowers the number of nonvolatile particles larger than 14 nm, leading to nvPM number emissions close to background concentrations. We therefore introduce a detection limit as a signal greater than the mean nvPM background concentration plus one SD to assess the significance of the data (Figs. 2 and 3). The detection limit varies depending on the homogeneity of background concentrations. In Fig. 3, data below the detection limit are nevertheless displayed but may be subject to a higher uncertainty due to the small difference to background deviations.

The comparison of lean and rich combustion modes shows a reduction of nonvolatile particle emission indices (EI_nv_) for particles larger than 14 nm by three orders of magnitude due to lean combustion, while total particle emission indices (EI_t_) for particles larger than 5 nm remain, considering measurement uncertainties, in the range of those from forced rich-burn mode. The reduction of the fuel aromatic and sulfur content down to close-zero values, characteristic for 100% HEFA-SPK, affects both EI_t_ and EI_nv_ compared to the reference Jet A-1: The number of total particles larger than 5 nm decreased by 65% in forced rich-burn and by 70% in lean-burn mode, likely due to the lower fuel sulfur content. Nonvolatile particle numbers were reduced by 64% in forced rich-burn mode and by 25% in lean-burn mode, consistent with the lower aromatic and higher hydrogen content of the HEFA-SPK fuel (see Fig. 2 and table S1).

Particle emission measurements of the A321neo test aircraft are shown in Fig. 3. In addition to the reference Jet A-1, a blended fuel containing 19% Jet A-1 and 81% HEFA-SPK as well as two HEFA-SPK with added aromatics (SPK + LA and SPK + HA) were tested, see Table 1. As already observed for the A319neo, EI_t_ remain similar for rich and lean combustion in the near field, while EI_nv_ are reduced by three orders of magnitude.

In forced rich-burn mode, we find that the fuel aromatic content, or the fuel hydrogen content, can determine EI_nv_. Measurements using SPK + HA show nvPM numbers similar to those of the reference Jet A-1, potentially because its high aromatic content leads to a hydrogen content similar to that of the Jet A-1. Combustion of the SPK + LA with a lower aromatic content leads to a reduction in EI_nv_ of 42% compared to the reference Jet A-1, and the blend fuel with the highest hydrogen content of 15.1% decreased nvPM numbers by 48%. When operating in lean-burn mode, nonvolatile particle emissions are close to the detection limit, which increases the uncertainty. Nevertheless, the data show a similar fuel dependence as in rich-burn mode, although nvPM is increased for the blend fuel, which could potentially be caused by its higher naphthalene content or contributions from background particles. For data below the detection limit, ambient aerosols can result in an apparent nonvolatile background-particle EI on the order of 10^11^ particles per kilogram of fuel burned at a distance of 30 to 250 m behind the source aircraft.

In both lean-burn and forced rich-burn mode, SPK + LA shows the lowest EI_t_ due to its low aromatic, naphthalene, and sulfur content. Higher fuel aromatic (SPK + HA) and sulfur contents (blend fuel) result in increased total particle emissions, with Jet A-1 showing the highest number of total particles. Despite similar EI_nv_, the number of total particles larger than 5 nm is reduced for the SPK + HA compared to the Jet A-1. The number of volatile particles may be determined by the chemiion concentration (*37*). However, the particle size and therefore the detectability by the CPCs are limited by condensable material in the exhaust plume. Therefore, the reduction in total particles could be attributed to the extremely low sulfur content of the fuel limiting the growth of volatile particles.

In summary, lean combustion reduced EI_nv_ by three orders of magnitude compared to the forced rich-burn mode for both tested LEAP-1A engines, while EI_t_ remained largely unaffected. Volatile particles continue to grow during the first minutes of plume evolution (*37*), and a fraction may not have reached the 5-nm cutoff size at 0.1- to 1.4-s plume age. EI_t_ could therefore be different for later stages of plume development, showing differences between rich and lean combustion that are not yet visible in the near field. For both combustion modes, HEFA-SPK and its blends decreased volatile and nonvolatile particle emissions. Thereby, a higher hydrogen/lower aromatic content of the fuel decreased nonvolatile particle numbers. The fuel sulfur content modulates the number of volatile particles, with aromatic compounds or engine oil potentially increasing the concentrations of hydrocarbon material in the plume, contributing to additional (semi-)volatile organic material.

### Engine-dependent variations in particle emissions

**Fig. 4.**
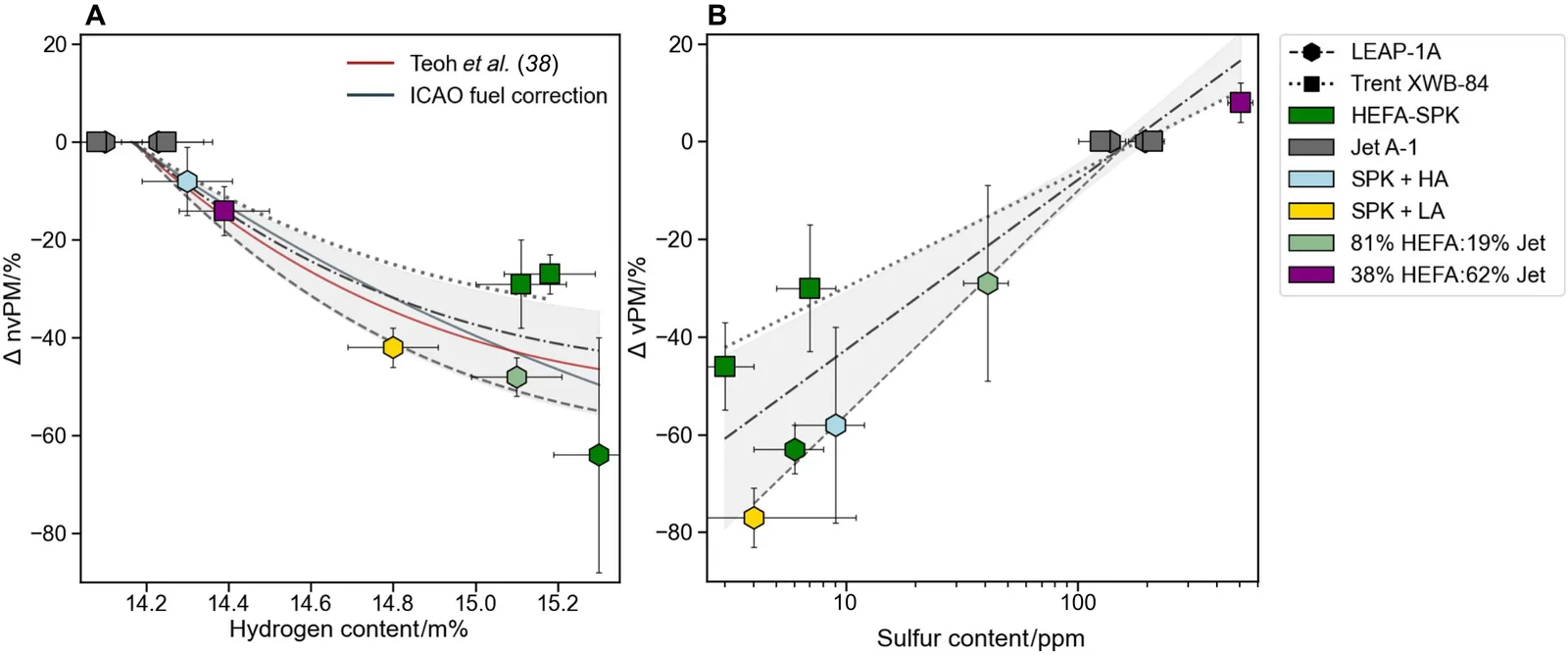
Mean difference in particle numbers as measured in near field due to the use of alternative fuels compared to Jet A-1 for rich-burn combustion mode; symbols show data points and lines show theoretical fits. (**A**) The difference in the number of nonvolatile particles >14 nm correlates exponentially with the hydrogen content of the fuel. (**B**) The difference in the number of volatile particles >5 nm, derived from the difference of nonvolatile and total particles, shows a logarithmic relationship with the fuel sulfur content. The magnitude of the differences in particle numbers varies between the CFM LEAP-1A engines and the Rolls Royce Trent XWB-84 engine [data from (*21*, *23*)] due to differences in engine design and potentially in the detailed composition of the fuels. Data show mean values for different fuel types color-coded, including SDs, as well as (A) exponential and (B) logarithmic interpolations covering data from both engines (dash-dotted); the corresponding 95% confidence intervals are shaded in gray. Dotted and dashed lines represent separate interpolations of data from the Trent XWB-84 engine and the LEAP-1A engine, respectively. (A) additionally shows the ICAO fuel correction for nvPM as a solid blue curve (*39*) and a correction proposed in (*38*) as a solid red curve. See the “Regression of particle number reductions” section for further details.

Figure 4 shows the dependence of the mean difference in particle numbers with respect to fuel hydrogen and sulfur content compared to the reference Jet A-1 of the respective measurement campaign. The data include near-field measurements from CFM LEAP-1A engines from the NEOFUELS/VOLCAN campaign series, as well as from Rolls-Royce Trent XWB-84 engines probed during the ECLIF3 campaign [data from (*21*, *23*)]. The engines operated in rich-burn (nominal operation for Trent XWB-84) and forced rich-burn mode (LEAP-1A). Weighted least squares (WLS) regressions were performed to quantify the relationship between the fuel parameters and the difference in particle numbers (see the “Regression of particle number reductions” section for formulas and more details).

Figure 4A shows the difference in EI_nv_ as a function of fuel hydrogen content. For both engines, EI_nv_ decreases quasi-exponentially with increasing fuel hydrogen content, where the applied expression follows an approach proposed in (*38*), see the “Regression of particle number reductions” section. Additional measurements with fuels containing 14.4 to 14.8% and <14.2% hydrogen would be valuable to reinforce the exponential relationship (*18*, *22*).

Fuels with similar hydrogen content result in larger nvPM reductions when burned by the LEAP-1A engine in forced rich-burn mode in comparison with the Trent XWB-84 engine. Both the ICAO fuel correction for nvPM (*39*) and the correction proposed in (*38*) represent the measurement data well, with increasing convergence toward LEAP-1A data for high hydrogen contents.

At present, Jet A-1 may contain up to 50% HEFA-SPK (*40*). ASTM is now developing a 100% drop-in SAF specification, which would permit increasing the SAF content in Jet A-1 up to 100% while remaining within existing fuel specification limits. Considering selected high-level fuel properties, such as density and aromatic content, SPK + HA is expected to fall within the specification limits; the remaining alternative fuels fall outside current certification standards due to their low aromatic content. Aromatic compounds play a critical role in ensuring proper seal functionality within aircraft fuel systems. The aromatic content is inversely related to the fuel hydrogen content, as the molecular ring structures of aromatic hydrocarbons contain fewer hydrogen atoms per carbon atom than the chain-like molecular structures of n-alkanes and iso-alkanes, which constitute the major fraction of hydrocarbons in HEFA-SPK. Chemical double bonds lead to higher bonding energies of the cyclic aromatic molecules, which therefore may represent more efficient soot precursors.

Figure 4B shows the difference in volatile particles >5 nm with respect to the fuel sulfur content. The number of volatile particles was obtained by subtracting nvPM numbers (*D*_p_ > 14 nm) from total PM numbers (*D*_p_ > 5 nm). Although most nonvolatile particles should be larger than 14 nm (*22*), the different cutoff sizes of the particle counters introduce an additional uncertainty. The number of volatile particles >5 nm (EI_v_) shows an approximately logarithmic correlation with the fuel sulfur content for both engines in the rich-burn mode. However, particularly for low-sulfur fuels, emitted compounds such as unburned hydrocarbons and lubrication oil potentially also contribute to the size and number of the volatile particles (vPM), causing a larger variation. The larger reduction in vPM for SPK + LA compared to SPK + HA, for example, could indicate that unburned hydrocarbons are an important contributor to vPM formation for low fuel sulfur contents. As observed for nonvolatile particles, the reduction in volatile aerosols due to the use of cleaner fuels may vary with engine type and the associated technology, with the LEAP-1A engine in forced rich-burn mode showing larger reductions compared to the Trent XWB-84 engine. Possibly, differences in soot particle effective diameter and primary particle sizes (*20*) between both engines could affect the uptake of volatile compounds by soot.

**Fig. 5.**
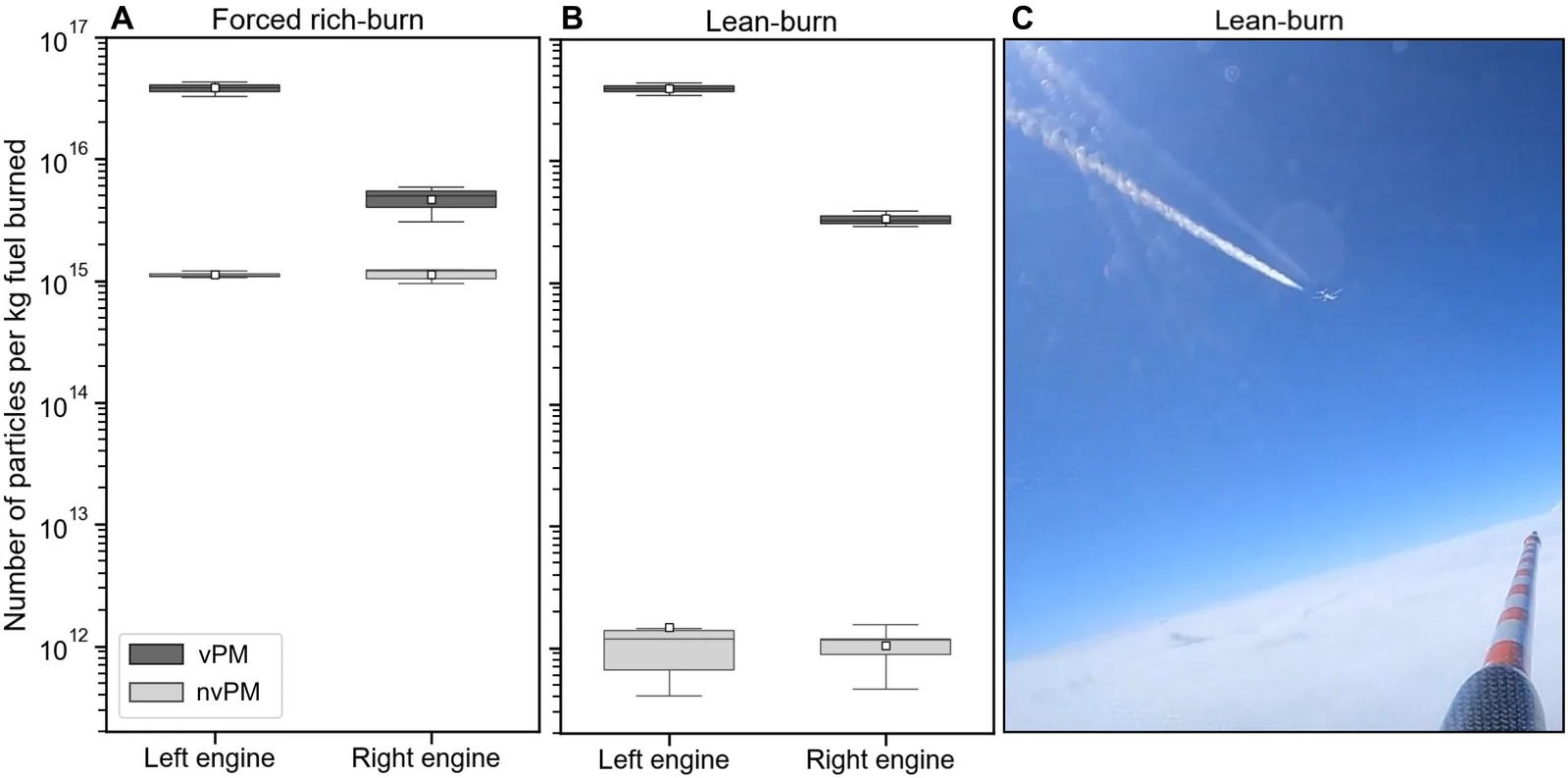
Engine-to-engine comparison of both LEAP-1A engines burning Jet A-1 in lean-burn and forced rich-burn during NEOFUELS/VOLCAN step 2. The number of volatile particles (dark gray boxes) and nonvolatile particles (light gray boxes) of the left-hand engine and subsequently the right-hand engine were measured in the near field in short succession in comparable atmospheric conditions for (**A**) lean-burn and (**B**) forced rich-burn operation. (**C**) View from the cockpit of the Falcon following the A321neo operating in lean-burn mode on a different day in contrail onset conditions. A lower optical thickness of the contrail behind the right-hand engine is visible. Data previously used in (*41*).

During one test flight conducted as part of NEOFUELS/VOLCAN step 2, a comparison of the emissions from the two LEAP-1A engines of the A321neo was performed. During the measurements, both engines were operated at similar medium cruise engine power settings and burning Jet A-1. First, the emissions from the left-hand engine were measured in both combustion modes, directly followed by measurements of the right-hand engine. Hence, the measurements were conducted in comparable atmospheric conditions. Figure 5 shows the number of volatile particles and nonvolatile particles per kilogram fuel burned of the forced rich-burn mode and the lean-burn mode. Both engines show similar EI_nv_ within measurement uncertainty in both forced rich-burn and lean-burn mode for similar T30 engine settings. In contrast, EI_v_ of the left-hand engine exceed EI_v_ of the right-hand engine by one order of magnitude.

The left-hand engine in this study exhibits an engine performance deterioration of 50%, while the right-hand engine shows a deterioration of 30% (see the “Source aircraft engines” section). These values indicate that the left-hand engine has experienced more severe wear or potentially degradation. In addition, the left-hand engine consumed ∼20% more oil, which is within the expected variation, although it may still indicate a possible systematic difference. This additional oil may contribute to an increase in volatile particle numbers and size compared to the right-hand engine. However, engine-to-engine variability within the fabrication limits could also contribute to the observed differences.

The difference between the two engines can also be observed visually when examining the contrail in lean-burn mode (Fig. 5C). In the soot-poor regime (≲10^13^ soot particles per kilogram of fuel), volatile aerosols likely contribute substantially to the formation of contrail ice crystals (*20*, *31*). The contrail behind the right-hand engine shows a lower optical thickness than the contrail behind the left-hand engine, indicating that fewer contrail ice particles developed behind the right-hand engine due to the lower number of volatile particles (*41*). While the operating parameters of both engines were similar and the effect persisted throughout the observation period, further investigations are necessary to confirm its persistence, temporal variations, and the range of oil consumption and to evaluate its dependence on engine settings and atmospheric conditions.

## DISCUSSION

As intended by the design of the LEAP-1A engine, lean combustion reduces emissions of nonvolatile particles (i.e., soot particles larger than 14 nm, corresponding to the CPC detection size range) by three orders of magnitude compared to forced fuel-rich combustion in noncontrail forming cruise conditions, bringing nvPM numbers in the exhaust plume close to background concentrations. With mean values of 1 to 6 × 10^11^ nonvolatile particles per kilogram of fuel burned, the measured EI_nv_ from lean combustion remain even below the lower bounds frequently assumed in contrail models (*16*, *20*). Depending on the aerosol background concentration, EI_nv_ below 1 to 3 × 10^11^ particles per kilogram of fuel burned can no longer be distinguished from background nvPM. For lean combustion emissions of clean fuels, such as the SPK + LA used in this study, ambient aerosols passing through the combustor and the engine bypass stream, or entrained downstream of the exhaust plane into the engine plume, can therefore result in a lower detection limit. This corresponds to an apparent nonvolatile background-particle EI on the order of 10^11^ particles per kilogram of fuel burned at a distance of 30 to 250 m behind the source aircraft and depending on factors such as plume dilution, ambient background concentrations, and measurement geometry.

Total particle numbers in the 0.1- to 1.4-s old exhaust are in the same range for both combustion modes, indicating only minor changes in concentrations of volatile aerosols >5 nm for the lean combustion mode of the tested engines. However, volatile particles continue to grow during the first minutes after formation (*37*, *42*). Hence, potential differences in volatile particle numbers between combustion modes might be observed at later stages of plume development, when the volatile particles have grown into the detectable size range >5 nm of the particle counter instrument. The reduction of EI_t_ and EI_nv_ through the use of alternative fuels correlates with fuel composition in lean-burn and rich-burn mode. Cleaner fuels with respect to hydrogen, aromatic, and sulfur content decrease particle numbers in rich-burn combustion mode, as supported by previous studies [e.g., (*18*, *21*–*24*, *43*, *44*)]. Here, we additionally demonstrate substantial particle reductions for SPKs for lean-burn engine configurations, as indicated for selected contrail conditions by Voigt *et al.* (*31*). Among the tested fuels, the combustion of conventional Jet A-1 shows the highest particle numbers due to its high aromatic and sulfur content. The combustion of nearly aromatic-free HEFA-SPK fuel results in the lowest nonvolatile particle numbers, reducing EI_nv_ up to 64% in forced rich-burn mode. According to engine models (*26*), the reduction could be larger when starting with a reference Jet A-1 with a world average fuel hydrogen content of 13.8%. During our campaign, the cleaner fossil kerosene supplied by the refinery to the Airbus flight testing site in Toulouse had a hydrogen content of 14.1%, which supports lower environmental impacts and improved airport air quality. Adding aromatics to HEFA-SPK increases EI_nv_. Volatile particle numbers correlate with fuel sulfur content in both rich-burn and lean-burn combustion modes, with reductions in volatile aerosol >5 nm of up to 80% for quasi-sulfur–free fuels.

The extent of the reduction varies with engine type and is likely not attributed to fuel composition alone. In our study, the CFM LEAP 1-A engine shows a larger reduction in the fuel-rich combustion mode through the combustion of cleaner fuels than the Rolls-Royce Trent XWB-84 engine for both volatile and nonvolatile particle EI. Although the particle number concentrations measured in the forced rich-burn mode are comparable to those observed for a RQL engine (*21*, *23*, *25*), particle properties may differ due to the distinct design characteristics of the two engine types, see the “Source aircraft engines” section. We note that the LEAP-1A combustor, when operated in the forced rich-burn mode, is not fully representative of an actual rich-burn combustor. This comparison should not be interpreted as a direct performance assessment of the two engines but rather as an illustration that reductions in particle emissions due to alternative fuels are not uniform. Instead, the reductions depend on engine and combustor design. Given the diversity of combustor and engine types of the global aircraft fleet, and the variation in fuel composition delivered to the airports in different continents, fuel-induced particle reductions should be viewed as a range rather than a single representative value.

Moreover, two engines of the same type but with different maintenance status may exhibit substantial variations in EI_v_. In our study, the number of volatile particles of the engine with greater deterioration was increased by a factor of 10. It should be noted, however, that such differences may also be influenced by inherent engine-to-engine variability, leading to subtle modifications of engine conditions. Given this observed variability, further investigations of volatile particles are recommended, as these are recently not reported to the ICAO Aircraft Engine Emissions Databank (*29*) and therefore largely unknown.

Reducing particle emissions will remain crucial for improving air quality and mitigating the climate impact of contrails in the future. As soot emissions decrease through the use of modern engines and alternative fuels, volatile particles are becoming increasingly relevant for contrail ice particle formation (*20*, *31*, *45*), and their reduction might also be targeted. Results from previous campaigns exploring the impact of fuel sulfur content on particle emissions and contrails (*37*, *46*–*52*) may have been obscured, as volatile particle effects might have been masked by the larger impact of nonvolatile particle emissions for rich-burn or RQL combustor configurations emitting in the high-soot regime (*18*, *25*). The relationships between particle emissions and fuel properties derived in this study provide a basis for modeling the associated health and climate impacts and for scaling particle emission models to cruise conditions.

For conventional jet fuels with median sulfur concentrations of 200 to 400 ppm (*34*, *35*), sulfur constitutes a major contributor to the formation of volatile particles in the aircraft exhaust along with contributions from organic compounds. Reducing fuel sulfur contents might thus contribute to both the mitigation of contrail climate impact (*21*, *31*) and the improvement of airport air quality (*2*, *53*). However, regulatory measures on fuel composition must also account for the potential trade-offs (*13*, *14*), such as reductions in shortwave radiative cooling associated with sulfate aerosol-radiation interactions and decreased albedo of low-level clouds (*54*, *55*).

Our findings demonstrate that a substantial reduction of particle emissions requires a synergistic approach, combining the use of alternative fuels with modern engine technologies. Also, the engine maintenance condition and engine-to-engine variability may have a notable impact on particle numbers and warrant further investigation. Last, engine design modifications on the lubrication oil venting might be considered, as lubrication oil released to the center core of the engine could form additional volatile particles (*45*, *56*), affecting contrail formation, particularly for current lean-burn engine configurations (*31*). Additional experimental studies investigating a broader range of engines and fuels are necessary to advance mitigation strategies and modeling approaches. In particular, targeted measurements and modeling of volatile aerosol properties and their impact on contrail ice crystal formation are needed to support the development of evidence-based regulatory frameworks for cleaner and competitive future aviation.

## METHODS

### Aerosol and trace gas measurements

Aerosol instrumentation included five butanol-based condensation particle counters (CPCs) (*18*, *57*, *58*) connected to a forward-facing isokinetic aerosol inlet. The CPCs, based on TSI CPC models 3010 and 3768a (TSI Inc., Minneapolis, MN, USA), were modified to operate in low-pressure environments. Three CPCs measured nvPM number concentrations by sampling through a heated inlet line of a thermal denuder at 250°C, while the remaining two measured tPM. The CPCs measuring nvPM were operated with different lower 50% detection efficiency diameters (D_50_) to assess particle size. For tPM, the D_50_ value was set to 5 nm, controlled by the temperature difference between the saturator and condenser. For nvPM, one CPC had a D_50_ of 14 nm, and the other two used diffusion screens to achieve larger cutoff sizes of ∼35 and 85 nm (*57*). The accuracy of these cutoffs was validated through laboratory testing with Ag aerosol against a Faraday Cup Electrometer (GRIMM Aerosol Technik, Ainring, Germany), and correction functions were applied to adjust for pressure-related variations. The characterization and calibration of aerosol instruments, as well as uncertainty analysis for near-field measurements, are described in detail in (*23*). The uncertainty in particle number concentrations is largely attributed to the uncertainty in the low pressure correction functions and amounts to 7 to 13% for an air pressure of 250 to 350 hPa in the near field in the absence of contrail ice. Because of the small size of exhaust aerosols, typically less than 0.1 μm, inlet effects are negligible in noncontrail-forming conditions.

Two instruments were used for CO_2_ measurements: a high-frequency (≈10 Hz) nondispersive infrared gas analyzer (Licor-7000, LI-COR Inc., Lincoln, NE, USA) for in-plume and background detection, with an accuracy of approximately 0.2 ppm, and a slower but more stable wavelength-scanned cavity ring-down spectrometer (CRDS, Picarro G2401-m, Picarro Inc., Santa Clara, CA, USA) for background and absolute mixing ratio measurements, with an accuracy ranging from 0.5 to 740 parts per billion (ppb) (average 55 ppb). These instruments were repeatedly calibrated using commercial gas standards (*59*). Reactive nitrogen (NO*_y_*) was measured using a chemiluminescence detector (Eco Physics, TR780), whereby reactive nitrogen species NO*_y_* (NO_2_, HNO_3_, PAN, and related species) are converted to NO using a gold converter (*T* = 290°C) with hydrogen as a reducing agent (*59*).

### Calculation of EIs

To account for dilution and mixing with ambient air, the particle EI was calculated. The EI correlates particle concentrations to fuel consumption based on measured CO_2_ concentrations, which have a known and constant EI (*22*), and is expressed as the number of particles per kilogram of fuel burned

$$\text{EI}=\frac{RT_{0}}{p_{0}}\cdot \frac{\Delta N}{\left[M\left(C\right)+\alpha M\left(H\right)\right]\cdot \Delta {\text{CO}}_{2}}$$ (1)

$T_{0}$ and $p_{0}$ are standard temperature (273.15 K) and pressure (1013.25 hPa), *R* is the ideal gas constant, α is the hydrogen-to-carbon molar ratio of the fuel, *M*(C) and *M*(H) are the molar masses of carbon and hydrogen, and $\Delta {\text{CO}}_{2}$ and ΔN represent the background-subtracted peak areas of CO_2_ and particle concentrations. The uncertainty of the EI

$$\begin{array}{c} \text{ΔEI}=\\ \sqrt{{\left(\frac{\text{dEI}}{dn_{x}}\right)}^{2}\left(\Delta n_{x}^{2}+\Delta {\mathrm{bg}}_{n}^{2}\right)+{\left(\frac{\text{dEI}}{{\text{dCO}}_{2}}\right)}^{2}\left(\Delta_{\text{err}}{\text{CO}}_{2}^{2}+\Delta {\mathrm{bg}}_{{\text{CO}}_{2}}^{2}\right)+{\left(\frac{\text{dEI}}{\text{d}\alpha }\Delta \alpha \right)}^{2}} \end{array}$$ (2)

is composed of the total uncertainty of the particle measurements $\Delta n_{x}$ (see the “Aerosol and trace gas measurements” section), the SD of the aerosol background $\Delta {\mathrm{bg}}_{n}$ of about 5 to 10% of the background concentration, the SD of the CO_2_ background $\Delta {\mathrm{bg}}_{\text{CO}2}$ of about 0.1 to 0.2 ppmv, the uncertainty of the CO_2_ measurement $\Delta_{\text{err}}{\text{CO}}_{2}$ of 0.2 ppmv, and the error in the hydrogen to carbon ratio Δα of 0.1%. In the near field, the uncertainty arising from deviations in aerosol and CO_2_ background concentrations is negligible due to the strong increase in concentrations in the early plume; therefore, the uncertainty of the particle measurement is the largest contribution. Overall, this results in an uncertainty of the EI of about 10% for the near-field measurements (*23*).

### Source aircraft engines

The A319neo and the A321neo were equipped with LEAP (Leading Edge Aviation Propulsion) engines, using TAPSII (Twin Annular Premixing Swirler) technology, a modern combustor design with focus on low emissions. These combustors premix fuel and air before combustion, resulting in a more uniform flame. In addition, the fuel is distributed through multiple injection points, enhancing the mixing process. Improved premixing reduces hotspots, which lowers NO*_x_* formation near stoichiometric conditions, and also suppresses soot formation by minimizing fuel-rich zones (*60*).

During certain test points of the flight test campaign, the combustor was additionally operated in a forced rich-burn mode, which was enabled by a temporary change of the fuel distribution logic commanded by the FADEC (full authority digital engine control). This temporary operating mode enriches the fuel-air mixture beyond the lean configuration by changing the injection location of the major part of the fuel flow. Usually, this rich-burn mode is used during transient conditions like engine startup, rapid throttle adjustments, or low-power settings, to ensure combustion stability and prevent flameout, while the combustor switches to lean-burn mode during stable operation at higher power (*61*). For the NEOFUELS/VOLCAN flight test measurements (*31*), the LEAP engine was operated in a forced rich-burn mode also during typical cruise flight conditions to investigate the impact of the different fuels also for fuel rich combustion. In contrast, today’s most commonly deployed RQL combustors, such as those used in the Rolls-Royce Trent XWB-84 engine, stage the combustion process by first operating in a rich-burn phase in the primary zone of the combustor, followed by rapid quenching and a final lean-burn phase. The initial rich-burn phase inherently leads to soot formation due to incomplete combustion in the fuel-rich zone (*23*, *62*, *63*). In the LEAP-1A, ∼70% of the compressor exit airflow is injected into the front zone of the combustor, whereas in RQL combustors only about 25% of the airflow initially enters the combustor primary zone, with further dilution occurring downstream (*64*). The air injection at different locations in RQL combustors leads to soot formation in the initial fuel/air mixing zone, followed by soot consumption downstream in the combustor as additional air is mixed in. The different flow field in LEAP-1A combustors may therefore alter the soot production-to-consumption ratio.

The left-hand engine of the A321neo in this study exhibits an engine deterioration of 50%, while the right-hand engine shows a deterioration of 30%, as determined by the engine manufacturer from the exhaust gas temperature (EGT) margin. Engine performance deterioration refers to the gradual decline in performance with accumulated flight hours and cycles, caused by mechanical wear, thermal stress, and environmental exposure. This results in reduced compressor and turbine efficiencies, which are compensated by increased fuel flow, rotational speeds, and therefore higher EGT. Engine maintenance and overhaul can partly recover the declining performance (*65*). Deterioration is often quantified in percentage terms, representing the relative loss in performance compared to a clean, nondegraded engine under standard operating conditions, where 0% deterioration corresponds to a new engine. In addition, inherent engine-to-engine variability can lead to differences in particle emissions and oil consumption. The oil consumption of the LEAP-1A test engines was on the order of 110 to 140 mg per kilogram of fuel burned. This is consistent with the 110 mg per kilogram of fuel reported in test-bench measurements across different engine types (*66*). For the LEAP-1A engine, a fraction of the nonrecovered oil is vented into the hot core exhaust stream. The left-hand engine tested during VOLCAN step 2 consumed about 20% more oil than the right-hand engine. Comparisons of particle emissions from different fuels and combustion modes were always conducted behind the right-hand engine. In addition, one measurement flight of NEOFUELS/VOLCAN step 2 included an intercomparison between the left-hand and the right-hand engine for the combustion of Jet A-1.

### Regression of particle number reductions

A WLS regression was performed to quantify the relationship between the fuel hydrogen content and the reduction in nvPM, as well as the relationship between the fuel sulfur content and the reduction in vPM (see Fig. 4). The weighting is based on the inverse of the squared uncertainties of the test points to account for heteroscedasticity in the data.

To represent the relationship between fuel hydrogen content and the percent difference in nonvolatile particle numbers ΔnvPM, we use an approximation proposed in (*38*). The initial model in (*43*) suggests a linear decrease in EI_nv_ for fuel hydrogen contents between 13.8 and 14.4%. The model in (*38*) extends this range toward higher hydrogen contents proposing the relation

$$\Delta {\text{nvPM}}_{13.8}\left(H_{\text{cont}}\right)\left[\%\right]=\left(\alpha_{0}+\alpha_{1}\cdot \hat{F}\right)\cdot \Delta H\cdot \text{exp}\left[0.5\cdot \left(\alpha_{2}-\Delta H\right)\right]$$ (3)

where a negative value corresponds to a reduction in nvPM number emissions due to the use of an alternative fuel with hydrogen content $H_{\text{cont}}$ (in percent) compared to a reference fuel with a hydrogen content of 13.8%; $\Delta H=H_{\text{cont}}-13.8$ denotes the deviation of the fuel hydrogen content from the reference value, $\alpha_{0}=-114.21$ and $\alpha_{1}=1.06$ are coefficients introduced in (*43*), $\alpha_{2}=0.5$ is a coefficient introduced in (*38*), and $\hat{F}$ is the engine thrust in percent. For flight conditions during near-field measurements, $\hat{F}$ is set to 45%, based on the approximation by Teoh *et al.* (*38*), with possible small variations between test points and between the VOLCAN and ECLIF3 campaigns.

Using Eq. 3, the remaining nvPM emission fraction (postreduction emission level) is given by

$$P\left(H_{\text{cont}}\right)\left[\%\right]=100+\Delta {\text{nvPM}}_{13.8}\left(H_{\text{cont}}\right)$$ (4)

During the measurement campaign, the mean hydrogen content of the reference Jet A-1 fuels was 14.17% and thus higher than the reference hydrogen content of 13.8% used by Teoh *et al.* (*38*). Consequently, Eq. 3 has to be renormalized with respect to the postreduction emission level at $H_{0}=14.17$, such that all reported changes are relative to the experimental baseline. We therefore introduce the renormalized reduction measure

$$\begin{array}{c} \Delta {\text{nvPM}}_{14.17}\left(H_{\text{cont}}\right)\left[\%\right]=\\ \frac{P\left(H_{\text{cont}}\right)-P\left(H_{0}\right)}{P\left(H_{0}\right)}=\frac{\Delta {\text{nvPM}}_{13.8}\left(H_{\text{cont}}\right)-\Delta {\text{nvPM}}_{13.8}\left(H_{0}\right)}{100+\Delta {\text{nvPM}}_{13.8}\left(H_{0}\right)} \end{array}$$ (5)

The resulting relationship is shown in Fig. 4A as solid red curve. In a second step, $\alpha_{2}$ was treated as a free parameter to capture the variability among the different engines. Resulting fit parameters and the coefficient of determination *R*^2^ of the fitting of the complete dataset, as well as those from fitting the individual engine types, are shown in Table 2.

**Table 2.** Fit parameters including SD and the coefficient of determination *R*^2^ for the WLS regressions of ΔnvPM and the fuel hydrogen content.

Engine	α_2_	*R* ^2^
All data	0.37 ± 0.18	0.88
LEAP-1A	0.74 ± 0.07	0.98
Trent XWB-84	0.00 ± 0.14	0.97

Figure 4A additionally shows an adaptation of the ICAO fuel correction for nvPM (*39*)

$$k_{\text{ratio}}\left(H_{\text{cont}}\right)=\text{exp}\left\{\left(0.99\hat{F}-1.05\right)\left(H_{\text{cont}}-14.17\right)\right\}$$ (6)

for a reference Jet A-1 with a mean hydrogen content of 14.17% and an alternative fuel with hydrogen content $H_{\text{cont}}$. The corresponding percent difference is given by

$$\Delta {\text{nvPM}}_{14.17}\left(H_{\text{cont}}\right)\left[\%\right]=100\left(k_{\text{ratio}}-1\right)$$ (7)

To represent the nonlinear relationship between fuel sulfur content and the percent difference in volatile particle numbers ΔvPM as shown in Fig. 4B, a WLS regression was applied using a linear fit to the natural logarithm of the sulfur content $S_{\text{cont}}$ as the independent variable

$$\mathrm{\Delta vPM}\left[\%\right]=a+b\cdot \text{log}\left(S_{\text{cont}}\right)$$ (8)

Fit parameters *a* and *b*, as well as the coefficient of determination *R*^2^ of the fitting of the complete dataset, as well as those from fitting the individual engine types, are shown in Table 3.

**Table 3.** Fit parameters including SD and the coefficient of determination *R*^2^ for the WLS regressions of ΔvPM and the fuel sulfur content.

Engine	*a*	*b*	*R* ^2^
All data	−77.47 ± 12.34	15.11 ± 2.43	0.84
LEAP-1A	−101.80 ± 3.13	19.90 ± 0.88	0.99
Trent XWB-84	−53.47 ± 3.99	10.25 ± 0.91	0.98
