# Supplementary material for: Mitigating aviation’s environmental impact through alternative fuels and cleaner engines
Source: Sci Adv. 2026 Sep 18;12(38):eaeg7503. doi: 10.1126/sciadv.aeg7503 (PMC13588160; doi:10.1126/sciadv.aeg7503)
Supplement: Supplementary file 1 [file sciadv.aeg7503_sm.pdf]

Science Advances
AAAS

Supplementary Materials for

Mitigating aviation's environmental impact through alternative fuels and cleaner engines

Rebecca Dischl *et al.*

Corresponding author: Rebecca Dischl, rebecca.dischl@dlr.de

Sci. Adv. **12**, eaeg7503 (2026)
DOI: 10.1126/sciadv.aeg7503

This PDF file includes:

Tables S1 and S2

5 Supplementary information

5.1 Particle emission indices

Table S1: **Mean values of particle emission indices.** Mean EI_{nv} and EI_t including standard deviation from particle measurements behind the A319neo (as shown in Fig. 2) and the A321neo (as shown in Fig. 3) operating in forced rich-burn or lean-burn combustion mode.

Aircraft	Combustion mode	Fuel	Flight date	Sampling time	EI_{nv} / kg^{-1}	EI_t / kg^{-1}
A319neo	forced rich-burn	Jet A-1	15 Nov 2021	104 s	$(7.6 \pm 2.5)\times10^{14}$	$(1.4 \pm 0.1)\times10^{15}$
A319neo	forced rich-burn	HEFA-SPK	15 Nov 2021	129 s	$(2.6 \pm 0.2)\times10^{14}$	$(0.5 \pm 0.1)\times10^{15}$
A319neo	lean-burn	Jet A-1	15 Nov 2021	223 s	$(2.5 \pm 1.1)\times10^{11}$	$(1.1 \pm 0.4)\times10^{15}$
A319neo	lean-burn	HEFA-SPK	15 Nov 2021	131 s	$(1.4 \pm 0.4)\times10^{11}$	$(0.4 \pm 0.1)\times10^{15}$
A321neo	forced rich-burn	Jet A-1	7, 11, 16 Mar 2023	763 s	$(8.8 \pm 0.8)\times10^{14}$	$(2.4 \pm 0.8)\times10^{15}$
A321neo	forced rich-burn	SPK+LA	11 Mar 2023	254 s	$(5.1 \pm 0.3)\times10^{14}$	$(0.9 \pm 0.1)\times10^{15}$
A321neo	forced rich-burn	SPK+HA	16 Mar 2023	269 s	$(8.1 \pm 1.0)\times10^{14}$	$(1.5 \pm 0.3)\times10^{15}$
A321neo	forced rich-burn	blend fuel	7 Mar 2023	392 s	$(4.6 \pm 0.3)\times10^{14}$	$(1.6 \pm 0.4)\times10^{15}$
A321neo	lean-burn	Jet A-1	7, 11, 16 Mar 2023	689 s	$(6.1 \pm 5.5)\times10^{11}$	$(3.2 \pm 1.7)\times10^{15}$
A321neo	lean-burn	SPK+LA	11 Mar 2023	262 s	$(1.2 \pm 0.6)\times10^{11}$	$(0.5 \pm 0.1)\times10^{15}$
A321neo	lean-burn	SPK+HA	16 Mar 2023	264 s	$(4.8 \pm 4.5)\times10^{11}$	$(0.7 \pm 0.1)\times10^{15}$
A321neo	lean-burn	blend fuel	7 Mar 2023	200 s	$(3.6 \pm 0.4)\times10^{11}$	$(1.1 \pm 0.2)\times10^{15}$

5.2 Flight conditions

Table S2: **Mean values of measurement conditions during near-field plume encounters.** Standard deviations of atmospheric parameters are shown in parentheses.

**Flight date**	**15 Nov 2021**	**7 Mar 2023**	**11 Mar 2023**	**16 Mar 2023**	**27 Mar 2023**
Figure	2a,b; 4	3; 4	3; 4	3; 4	5
Fuel	Jet A-1; HEFA-SPK	Jet A-1; Blend	Jet A-1; SPK+LA	Jet A-1; SPK+HA	Jet A-1
Aircraft	A319neo	A321neo	A321neo	A321neo	A321neo
Contrail age (s)	0.1–1.2	0.2–1.4	0.2–1.2	0.2–1.4	0.3–1.2
Flight Altitude (m)	9150	9140	9140	9140	8830
Ambient RHi (%)	N/A	72 (19)	65 (13)	47 (8)	65 (11)
Ambient T (K)	229	226	230	229	227
ΔT_{SA} (K)	5.1 (0.6)	2.1 (0.3)	4.7 (0.9)	4.9 (0.5)	0.9 (0.3)
Aircraft Speed (Mach)	0.62	0.59	0.59	0.59	0.58
